# Methicillin-Resistant *Staphylococcus aureus* (MRSA) and Other Methicillin-Resistant Staphylococci and *Mammaliicoccus* (MRNaS) Associated with Animals and Food Products in Arab Countries: A Review

**DOI:** 10.3390/vetsci9070317

**Published:** 2022-06-24

**Authors:** Chahrazed Belhout, Rachid Elgroud, Patrick Butaye

**Affiliations:** 1 HASAQ Laboratory, High National Veterinary School, Issad Abbes Avenue, Oued Smar, El Harrach, Algiers 16270, Algeria; 2 Institute of Veterinary Sciences, University Frères Mentouri Constantine 1, Constantine 25017, Algeria; elgrouddz@yahoo.fr; 3 Department of Pathobiology, Pharmacology and Zoological Medicine, Faculty of Veterinary Medicine, Ghent University, Salisburylaan 133, B9820 Merelbeke, Belgium; patrick.butaye@ugent.be

**Keywords:** methicillin-resistant staphylococci (MRS), methicillin-resistant *S. aureus* (MRSA), methicillin-resistant non-*aureus* staphylococci (MRNaS), methicillin-resistant coagulase-negative staphylococci (MRCNS), animals, food products, Arab countries, North Africa, Middle East, MENA

## Abstract

**Simple Summary:**

Staphylococci are present in the microbiota of both humans and animal species, being recognized as the most important opportunistic pathogens. Antimicrobial resistance (AMR) has become a global public health issue presenting a significant risk because it severely limits treatment options. Methicillin resistance in staphylococci (MRS) poses a specific problem as it may cause serious human and animal infections, eventually resulting in death. The increasing observation of MRS in different animal species has raised the concern of their impact on animal health and the potential of zoonotic transmission. The availability of comprehensive data on the ecology and distribution of MRS in animals and food products worldwide is necessary to understand their relevance in the “One Health” domain. However, there is a gap in information in terms of MRS and the Arab countries. Therefore, our study aimed to provide an overview of the situation of MRS in these countries by reviewing the available data on livestock and animal products and making recommendations for the future.

**Abstract:**

The prevalence of methicillin resistance in staphylococci has been increasing globally and is currently one of the major public health concerns. In particular, treating infections caused by staphylococci with acquired antimicrobial resistance is problematic, as their treatment is more difficult. The resistance is found both in human and animal staphylococcal strains. Methicillin-resistant staphylococci (MRS) have also been increasingly reported in wildlife. In Arab countries, MRS has been detected in food producing animals and food products; however, the risk this poses is somewhat unclear, and still a significant lack of information on the trend and distribution of these pathogens in these countries, which have a specific ecosystem (desert) and traditions (Muslim culture). In this manuscript, we aim to provide an overview of the prevalence and the major MRS clonal lineages circulating in these specific countries and compare to them other situations with different ecosystems and cultures.

## 1. Introduction

The genus *Staphylococcus* is currently composed of more than 84 recognized species and 30 subspecies [1]. Recently the genus has been reassessed and some species have been transferred to a new genus, *Mammaliicoccus*, which contains 5 former *Staphylococcus* species (Table 1) [2]. *Staphylococcus sciuri*, *Staphylococcus fleurettii*, *Staphylococcus lentus*, *Staphylococcus stepanovicii* and *Staphylococcus vitulinus* have been transferred to the novel genus *Mammaliicoccus* with *Mammaliicoccus sciuri* as the type species [2]. The staphylococci are divided into two distinct groups: the coagulase-positive staphylococci (CPS), such as *Staphylococcus aureus* and six other species, and the coagulase-negative staphylococci (CNS) [2,3]. They generally are part of the normal flora of mammals and birds [4], though, upon predisposing factors, they can cause mild to serious life-threatening diseases [5]. Some species are recognized as human and facultative animal pathogens, especially the coagulase-positive species though also some coagulase-negative species such as *Staphylococcus epidermidis*, *Staphylococcus haemolyticus*, *Staphylococcus lugdunensis*, and *Staphylococcus saprophyticus* are well known facultative pathogens [6,7], generally implicated in nosocomial infections [8].

Antimicrobial resistance (AMR) has become a global public health issue, presenting a significant risk because it severely limits treatment options. Almost all countries, irrespective of their wealth, are facing this threat [9,10]. Methicillin-resistant staphylococci (MRS) pose a specific problem, as they may cause serious human and animal infections, eventually resulting in death [11]. Methicillin-resistant *S. aureus* (MRSA) emerged within two years after the introduction of staphylococcal beta-lactamases resistant beta-lactams, with methicillin being the first introduced [12]. Acquisition of methicillin resistance is due to integration of the staphylococcal cassette chromosome *mec* (SCC*mec*), which contains the *mecA* gene conferring resistance to β-lactams [13]. In 2011, A divergent *mecA* homologue, *mecA*_LGA251_, later named *mecC*, was discovered and located in a novel staphylococcal cassette chromosome *mec* element, designated SCC*mec* type-XI [14]. The *mecC* was 70% identical to the *mec*A gene and was initially detected in 15 *S. aureus* isolates from dairy cattle in England [14].

Apart from *S. aureus*, methicillin resistance has also been discovered in many staphylococci and are named methicillin-resistant non-*aureus* staphylococci (MRNaS) [15,16,17]. MRNaS have been isolated from humans [18] and animals [17,19], and are proposed to be a potential reservoir of SCC*mec* elements for *S. aureus* [20].

The infections caused by MRSA were initially confined to hospitals and healthcare facilities and were named hospital-acquired MRSA (HA-MRSA), with sporadic infections in the community related to direct exposure to the healthcare system [21]. During the mid-1990s, new MRSA lineages emerged, which were, in general, quite susceptible to different antimicrobials and possessed the SCC*mec*IV [22]. The first infections with these new clones were found in native people in Australia and North America, as well as in people involved in contact sports [23]. These strains spread in the community and were likewise named community-acquired MRSA (CA-MRSA) [24]. During their evolution, they became dominant in hospitals [25]. In 2005, a new MSRA clone, ST398, was found in livestock [26], and was subsequently named LA-MRSA [27]. This clone was shown to have originated from humans and seemed to have lost host specificity while acquiring methicillin resistance [28]. Further studies showed the increasing diversity of LA-MRSA [29]. Humans in contact with livestock, mostly pigs, could be temporarily infected with these strains, suggesting a zoonotic transmission [30]; however, those clones represented little pathogenicity for humans in general [31].

**Figure 1:**
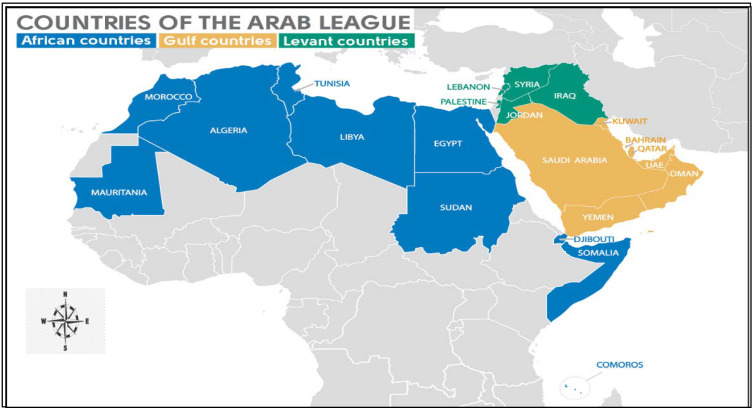
Map of the Arab world countries [34].

The Arab world that occupies the Middle East and North Africa (MENA), also called Arab Nations, consists of twenty-two Arabic-speaking countries of the Arab League with a population of about 420 million people (Figure 1) [32]. Ten countries in North Africa (Algeria, Egypt, Libya, Morocco, Sudan, Tunisia, Mauritania, Djibouti, Comoros and, Somalia) and twelve in the Middle East (Bahrain, Iraq, Jordan, Kuwait, Lebanon, Oman, Palestine, Qatar, Saudi Arabia, Syria, the United Arab Emirates, and Yemen) [33]. The Arab World has a different lifestyle than other nations. It is a region bound by tradition (Bedouin Society) and religion (Muslim culture). There are differences between these countries in terms of resources, growth rates, and economic strengths [34]. The Gulf countries (Saudi Arabia, Bahrain, United Arab Emirates, Kuwait, Oman and Qatar), for example, enjoy relative economic stability and wealth compared to other Arab countries [35].

There is no doubt that AMR is one of the most dangerous threats to public health in the world and it seems that the Arab countries present an ideal environment for the spread of resistant strains [31,36]. This could be related to several reasons, including the over/misuse of antibiotics in humans and animals, and the high mobility of animals and herders in some countries. Next to that, by large, infection control measures are absent. From a sociological standpoint, the continuous population mobilization due to socioeconomic conflicts and multiple war crises are to be taken into account [34,37]. Other factors involved may be the specific environmental conditions, with large deserts and limited places with water where people cluster together with their animals, as well as the intensification of animal production [38]. These specific regional characteristics may have a relevant impact on the epidemiology of MRS.

The problem with methicillin resistance in staphylococci in the Arab countries, as in other parts of the world, has grown steadily [39]. However, its extent is currently not clear since surveillance of drug resistance is only carried out in a few Arab countries [37,40]. Since no review summarizing the situation on MRS in Arab countries is yet available, we summarize and analyse the fragmented single-centered research publications. Therefore, in this manuscript, the objective is to review the current MRS situation (MRSA and MRNaS) in animals and food in Arab world countries.

## 2. Methicillin-Resistant Staphylococci (MRS) in Animals

### 2.1. MRSA

#### 2.1.1. MRSA in Food-Producing Animals

The first report of MRSA infections in food-producing animals was a case of bovine mastitis in Belgium in the early 1970s [41]; however, this was a strain of human origin and did not spread [42]. Since 2005, one of the most important events in the epidemiology of infectious diseases that has attracted considerable public health attention [43] is the emergence and spread of specific clones of MRSA in livestock, named Livestock-Associated MRSA (LA-MRSA) [27]. Subsequently, increasing numbers of reports have been published on LA-MRSA infection and colonization of both companion and food-producing animals, indicating a growing awareness of the role of these animals in the evolution, epidemiology, and dissemination of these microorganisms [44] and evidencing LA-MRSA as potential zoonotic pathogen though to a lesser extent an animal pathogen, as it has been shown only to be implicated in mastitis in bovines [45] and disease in turkeys [46]. However, LA-MRSA CC398 is thought to be less pathogenic for humans than other *S. aureus* lineages [47]. The initial LA-MRSA isolates belonged to clonal complex 398 (CC398), which was very uncommon in humans at the time [48]. Since its discovery, LA-MRSA CC398 has been isolated from cattle, horses, chickens, and turkeys, but currently, pigs appear to be its primary host [49]. The presence of LA-MRSA CC398 in food-producing animals is a matter of concern due to direct transmission to people in contact with infected animals and/or their products, as well as possible contamination of food [48]. Although CC398 is still the most common LA-MRSA worldwide, other sequence types are involved, such as CC9, CC97 and CC1 [29].

In Arab countries, research on MRSA in animals/food is quite limited and there is only a little information available from some countries (Table 2) [50] with obviously fewer data on pigs from Arab countries, as there is only a minority of people consuming pig meat (consumption of pork is a religious taboo). The major focus of EFSA surveillance in European countries is pigs, however, as they represent the main reservoir of LA-MRSA [51]. The voluntary monitoring of the member states for the prevalence of MRSA in food and food-producing animals in 2018–2019 revealed that the prevalence varied between animal species, production systems, as well as the country [51].

In pigs, the prevalences varied between 0.1% for pig herds in Norway and 100% for fattening pigs in Portugal. Most strains were LA-MRSA CC398 [51]. In cattle, moderate MRSA prevalence was reported among herds of dairy cows and in herds of meat-producing animals (14.0% and 8.7%, respectively) in Belgium and slightly lower in Denmark (6.1% and 1.5%, respectively), while the higher level was reported from herds of veal calves in 2019 (9.6% and 54.5%) in Denmark and Belgium, respectively [51]. In poultry, a low MRSA prevalence was reported among laying hen flocks in Denmark in 2018 (3.2%), and a moderate level was reported among fattening turkey flocks by Germany in 2018 (17.2%) [51]. The majority of the MRSA isolates recovered from pigs, cattle, and poultry flocks on which molecular typing data were reported in 2019, were livestock-associated lineages [51].

These data are high compared to those reported from the Arab countries; this could be related to the lack of studies and surveillance available in these countries.

##### MRSA in Cattle

In cattle, *S. aureus* is considered a contagious pathogen, causing clinical and subclinical mastitis [68,69,70]. Methicillin-resistant *S. aureus* has been reported from dairy farms worldwide and has been associated with transmission events between humans and animals [71,72]. In the Arab World, a comparison of MRSA prevalence studies is challenging because of differences in types of samples, inoculum volumes, pre-enrichment, and detection methods. Some studies used selective media and/or PCR confirmation for the isolation and confirmation of MRSA [57,59,73], while other studies determined the prevalence of phenotypic methicillin resistance in *S. aureus* [53,54]. Nevertheless, the prevalence of MRSA was, in general, rather high in milk samples and ranged between 3.60% [55] and 35.7% [54]. Carriage of MRSA in the noses of cattle has been reported to be in the range of 15.5 to 40% [52,58]. The higher prevalence of MRSA in cattle may be explained by the fact that intensive production systems are mostly used in cattle farming [74]. Moreover, the surveillance program that controls cattle importations and farm biosecurity measures for personnel visiting or returning from abroad are not sufficient or absent—this could represent the most important way of introducing MRSA.

Several MRSA lineages have been identified in specific geographical areas. In the Arab countries, MRSA ST97-t267 has been reported in healthy cows in Tunisia [60]. MRSA ST97 was first discovered in Italy in pigs [75]. ST97 and other related STs belonging to CC97 are traditionally closely associated with cattle and have been recently described in both healthy and diseased pigs in European countries [76]. In Egypt, CC1, CC5, and CC45 have been isolated from cattle [56]. CC1 and CC5 have been recognized as both HA- and CA-MRSA but have also been isolated from pork and pigs [77]. These CCs are highly prevalent in humans in Europe and the Middle East [78,79]. MRSA CC22 has been reported from cattle in Egypt [59] and is considered HA-MRSA. CC22 strains were first identified in the UK in the early 1990s and are the most prevalent HA-MRSA in the UK [80].

MRSA from cattle may represent a potential zoonotic issue, especially for people in direct contact with the animals, though also for the general public through the food chain. It is clear that in Arab countries, cattle are a significant reservoir for MRSA [81]; however, it still unclear what is the real burden of MRSA in cattle in Arab countries as few data have been published. There is a need for more research on the prevalence of MRSA and their genetic background to have a clear view of the burden of MRSA in cattle rearing as well as for public health.

##### MRSA in Sheep and Goat

Historically, in Arab countries—in contrast with European countries—sheep and goats dominate, and they are considered a crucial source of meat and milk for the human population in that area [82]. The high consumption of sheep and goat meat in these countries compared to other European countries can be explained by the mainstream religious and socioeconomic conditions in this area [83]. Typically, in North African countries such as Tunisia, Morocco, and Algeria, sheep herds are distributed all over the country on small family farms, though there are also large farms with intensively reared animals [84]. Frequently, unpasteurized ovine milk is used for the artisanal production of cheeses, which increases the risk of transmission of pathogens through milk [85]. The exploitation of goat herds for meat and milk is widespread around the world [86], with the largest proportion in Asia and Africa [87,88]. Goat meat is widely regarded as a lean meat, and there are indications that the demand for this healthy meat will increase [89]. 

Staphylococci have been isolated from various body sites, as well as from infections, mainly mastitis, from sheep and goats [90]. Nasal carriage of MRSA in sheep in Arab countries ranged between 3% and 28.9, respectively [52,62], while in milk samples, a prevalence of 29.8% has been reported in Jordan [57]. In goats, the highest prevalence of MRSA was 17.4% in milk samples [64], while a prevalence of 2% was detected using nasal swabs [63]. Only MRSA belonging to the CC80 has been found with ST80 and ST153 in Algeria and Tunisia, respectively [61,62]. ST80 is a typical CA-MRSA, first described in Europe but now spreading worldwide [91,92,93]. Mastitis in sheep and goats has been associated with different STs reported worldwide, such as ST291 [94], ST750, ST1729 [95], ST1 [96].

It is currently quite difficult to draw conclusions on the situation of MRSA in sheep and goats in Arab countries due to the lack of data, but current data indicate a rather high prevalence.

##### MRSA in Poultry

The growth of commercial poultry farming in Arab countries has provided a fertile field for staphylococcal infections and zoonotic transfer [97]. Its intensive nature engenders the extensive use of different antibiotic agents for treatment, imposing a considerable selection pressure for resistance. The first detection of LA-MRSA in poultry came from Belgium, where LA-MRSA was isolated from healthy broilers [98]. Consequentially, MRSA has been detected in poultry in several Arab countries [99]. Two studies from Algeria showed a prevalence between 30% [65] and 57% of MRSA in poultry [53] (Table 2). The prevalence of methicillin resistance among *S. aureus* was 27% in Egypt [66] and 27.3% in Iraq [67].

The majority of the MRSA isolates belonged to CC398 with a single CC5 isolated from a turkey, though it should be noted that typing has only been performed in a single study from Egypt [66]. The most frequent *spa* types were the typical animal-associated t011, t034, and t899 [66]. CC398 has previously been reported in several cases in poultry [100,101,102,103]. CC5 is commonly associated with human infections and has been isolated from pork and pigs in the USA [77] and Canada [104]. It has been shown that CC5 originally comes from humans and has been adapted to poultry, causing infections [105]. 

##### MRSA in Camelids

There are about 30 million dromedary camels in the world, with the highest number in Africa and the Middle East [106]. Particularly, in Arab world countries, the dromedary camel (*Camelus dromedarius*, one-humped camel) is a multipurpose animal and an important livestock species adapted to hot and dry environments [107], formerly used strictly for transport [108], but nowadays also production animal for milk, meat, and hides [109]. The consumption of camel milk is ten times more than that of its meat, and it is expected that milk production will double in the near future [110]. Camels were formerly thought not to be affected by many of the diseases commonly impacting livestock [111]. However, recent data have confirmed their susceptibility to a high number of pathogens [106], and camels are currently believed to act as a carrier or reservoir for the transmission of several transboundary animal diseases and zoonoses [106], such as Middle East respiratory syndrome virus (MERSV) [112] and prion disease [113].

While there are data on *S. aureus* and MRSA on camel-derived food products [114,115,116], there is only one study that confirms the presence of methicillin resistance in *S. aureus* in these animals [61]. The MRSA isolates belonged to the European community-associated CA-MRSA CC80, which could indicate that the origin of these strains is human as it is one of the most reported CCs in humans in the Arab world [117]. Further studies are necessary to determine the extent and potential zoonotic aspect of MRSA in camelids.

#### 2.1.2. MRSA in Other Animals

MRSA has also been detected in pet animals, pest animals and wildlife [118,119]. Generally, MRSA strains of these animals differ from those of livestock and production animals [21]. Different studies showed that MRSA strains isolated from companion animals are mainly of human origin and are passed between human owners and their animals due to intimate contact [120,121]. Likewise, they can pass these strains back to humans. However, in Arab countries, data on the prevalence and characteristics of MRSA in non-farm animals are scarce [122,123,124]. The prevalence of MRSA in pets ranged between 5.3% and 25% [122,123]. Only one study on bat guano found an indication of the presence of MRSA in wildlife. The MRSA were the typical human-associated CC5-ST149 Maltese clone [125]. By and large, the presence of MRSA in wildlife in Arab countries remains unknown.

#### 2.1.3. MRSA in Livestock-Derived Food Products

While the direct transmission of MRSA has been well studied, the role of MRSA on food is still poorly understood [126]. Staphylococcal foodborne disease (SFD) is a common food-borne toxi-infection resulting from the contamination of food by enterotoxins [127,128], of which many types have been found [129]. These enterotoxins function as superantigens, cause immunosuppression and trigger non-specific proliferation of T cells leading to high fever; the clinical symptoms of *S. aureus* food poisoning are, however, mostly relatively mild [130]. The number of foodborne illnesses caused by *S. aureus* is estimated to be much higher than those reported [130]. It has been shown that variation in the consumption of foods and food habits are the major causes of differences in the incidence of SFD [131]. Food handlers carrying enterotoxin-producing *S. aureus* in their noses or on their hands are regarded as the main source of food contamination, via manual contact or through respiratory secretions [132]. Because *S. aureus* does not compete well with indigenous microbiota in raw foods, contamination is mainly associated with improper handling of cooked or processed foods, followed by storage in conditions that allow for the growth of *S. aureus* and production of the enterotoxin [133]. In contrast, food poisoning caused by MRSA strains is very rare. The first outbreak of gastrointestinal illness caused by MRSA originates in the United States [134], where a colonized food handler was found to be the cause of the outbreak by contaminated coleslaw [134].

In Arab countries, MRSA has been reported to be present in different types of foods (Table 3), such as beef, poultry meat, raw cow’s milk, and camel and sheep milk [114,115,135,136,137,138,139,140,141], but if MRSA can act as a food-borne pathogen remains unclear. CA-MRSA, LA-MRSA and even HA-MRSA in foods have been reported in these countries [141,142], revealing both the human and animal origin of the strains [116].

Some ready-to-eat food products (turkey parts, hot meals, salads) have also been found to be contaminated with MRSA strains [142,143,144] and are likely to be a source of contamination and transmission of resistant strains that pose a risk to human health [143].

MRSA have been reported in Europe in meat samples from cattle, pigs, broilers, and turkeys during the 2018 and 2019 surveillance, though the prevalence varied between meats of different origins and ranged from very low (0.3%) to very high (100%) [51]. Notably, turkey meat tested in the Netherlands and Austria were all positive for MRSA [51]. Additionally, MRSA was detected in samples of raw cow’s milk in 2019 in Germany [51]. Overall, most of the MRSA isolates were typical LA-MRSA [51]. In general, the transmission of MRSA via the food chain is considered to be a minor transmission route to humans, and detection often involves selective culture techniques that may detect very low levels of contamination.

**Table 3:** Prevalence and types of MRSA in food products in Arab countries.

Source	Year	Country	No. of Tested Samples	No. of MRSA Positive	Lineages of MRSA	Reference
Dairy cattle, sheep, and goat bulk tank milk	2017	Jordan	208	54 (25.97%)	ND	[57]
Raw cow milk	2018	Tunisia	300	3 (20%)	ST4114 ST4120	[74]
Raw camel milk	2019	Saudi Arabia	100	10 (50%)	ND	[114]
Raw cow milk and traditional dairy products	2018	Algeria	270	11 (17,74%)	ST8	[135]
Chicken meat	2020	Egypt	144	8 (6%)	N/D	[136]
Raw chicken meat	2015	Tunisia	164	2 (1.2%)	ST30 t012 ST398 t4358	[141]
Retail meat (camel and chicken)	2016	Saudi Arabia	100	6 (6%)	CC1 CC15 CC80 CC88	[142]
Beef meat	2017	Egypt	100	4 (4%)	N/D	[145]
Unpasteurized milk sold	2021	Algeria	82	3 (30%)	ST80	[146]

N/D: Not determined.

##### MRSA on Meat and Meat Products

MRSA has been detected on beef, chicken, and camel meat in some Arab countries (Table 3). However, most of the studies were limited to phenotypic detection of methicillin resistance in *S. aureus*, and as such, the true prevalence of MRSA in meats are difficult to estimate. MRSA strains have been found in raw meat in Tunisia and Saudi Arabia [141,142]. Six sequence types have been identified (ST30, ST398, CC1, CC15, CC80, CC88) [141,142]. Most of these CCs are associated with humans, CA-MRSA (CC15, CC1, CC80, CC88, ST30), indicating that most of the MRSA on food are from food handlers. It was only in Tunisia where LA-MRSA ST398 was reported to be found on chicken meat [141].

##### MRSA in Milk and Milk Products

It is s well known that staphylococci, including methicillin-resistant strains, are an important cause of mastitis in dairy cows, ewes, buffalo and camels [114,147,148]. The bacteria are subsequently excreted into the milk without organoleptic alteration, allowing them to spread through the food chain if the milk is not treated properly [59,135,146,148,149]. MRSA has been reported in both raw and pasteurized milk from cattle and camels [115,135,149,150] (Table 3). The traditional dairy products, which occupy an important place in the diet in Arab countries and particularly in the North African countries (Algeria, Tunisia, Morocco), have also been shown to carry MRSA [151]. The MRSA types belonged to well-known human types (ST80, ST8, ST4114, ST4120) [74,135,149], though some, like the ST80 strains highly prevalent in the Mediterranean region, have also been detected in animals and food products [61,149]. The ST8 lineage, detected in raw milk in Algeria [135], is related to the USA300 clone, which is predominant in the United States, South America [152] and the Caribbean [153]. ST4120 belongs to CC5, frequently detected in human infections [154]. These MRSA were probably transmitted by humans, indicating that improving food hygiene is the solution to the problem.

In EFSA surveillance, there are data about the prevalence of MRSA in milk and milk products. MRSA was reported in raw cow’s milk in Germany and Denmark, with *mec*C-MRSA CC130 (spa-type t843) identified for the first time from bulk tank milk in Denmark [51].

### 2.2. MRNaS

Methicillin-resistant non-*aureus* staphylococci (MRNaS) are all staphylococci but *S. aureus*, resistant to β-lactam antibiotics [155]. For this review, we also included the former *Staphylococcus* species that have been reclassified as *Mammaliicoccus* species since they were only recently transferred to this new genus and were also shown to carry similar SCC*mec* elements as other staphylococci [15]. Methicillin resistance has been detected in nearly all staphylococcal species showing that SCC*mec* is likely more mobile than assumed [29]; however, there is not always a lot of information on them as in general, they cause less pathology and as such, are studied to a lesser extent. Nevertheless, given their abundance, they probably play an important role in the spread of methicillin resistance in staphylococci. The MRNaS species on which most information is available are methicillin-resistant *Staphylococcus epidermidis* (MRSE) [156] and methicillin-resistant *Staphylococcus pseudintermedius* (MRSP) [157,158]. MRNaS are of interest as they represent a reservoir of SCC*mec* [20]. Though indirectly based on epidemiological studies, the transfer of (parts of) SCC*mec* between MRNaS and *S. aureus* has been shown in several studies [30,159].

The available literature on MRNaS in animals and food is still very limited to non-existent in terms of most Arab countries. In Egypt, *S. epidermidis* and *S. warneri* strains obtained from goats and cattle (nasal swabs), respectively, were shown to harbor the *mec*A gene [160]. In addition, in *S. intermedius*, *S. cohnii*, *S. capitis*, *M. sciuri* [123] and *S. pseudintermedius* [124] from Libyan cats and dogs, the *mec*A gene has been detected [123]. On food products, *mec*A-positive *S. hyicus*, *S. intermedius*, *S. lugdunensis* have been detected [161]. Frequently those isolates were *mec*A positive but did not show phenotypic resistance [161]. There is clearly a need for more data so as to be able to determine the role of MRNaS in Arab countries.

## 3. MRS in Animals and Its Impact from a One Health Perspective

Today, the world is attempting to address a global pandemic of antimicrobial resistance and increasing problems with infections with multidrug-resistant bacteria, namely the bacteria of the ESKAPE group: *Enterococcus faecium*, *Staphylococcus aureus*, *Klebsiella pneumoniae*, *Acinetobacter baumannii*, *Pseudomonas aeruginosa*, and Enterobacteriaceae [162,163]. A holistic approach, One Health, is an important approach to preventing the emergence and spread of these resistant pathogens and maintaining the effectiveness of existing antibiotics [164]. One Health is a global health concept that emphasizes the interconnection of different ecosystems, focusing on humans and animals (pets, livestock and wildlife) and the environment [165]. Staphylococci are important in the One Health concept as some species and clones have been shown to have a multi-host ecology [28]. MRSA have a typical clonal population structure with single or multiple host tropisms [166,167], while the situation with MRNaS is generally less clear.

## 4. Conclusions

The present review shows major gaps in our knowledge of MRS (MRSA and MRNaS) in Arab countries. While some countries are Low- and Middle-Income Countries (LMICs) and thus, fewer data exist, there is also a lack of data from wealthier countries. Most of the eligible studies used in this current article were performed in North African countries when the high prevalence of MRS was reported. From the few data available, it is clear that animals can heavily be contaminated with MRS and can be an important component of the One Health spread of methicillin resistance. Several clonal lineages associated with animals have been identified in Arab countries, and the detection of MRSA CC398 and CC130 strains stand out. However, there is very scarce information about potential reservoirs and ways of dissemination of these clones in these countries. The current studies are also too fragmentary and use different methodologies; thus, they are not comparable with other studies. Hence, it is recommended that more holistic One Health studies on a global level (and not only involving high-income countries) are conducted to understand the burden of MRS. This will also increase our knowledge of the phylogenomic relationship between the strains and their evolution over time and can be a powerful tool for a better understanding of the epidemiology of this microorganism and for establishing appropriate control measures.

## Figures and Tables

**Table 1:** Recent reclassification of members of the genus *Staphylococcu*s (Adapted from [2]).

Staphylococcal Species and Subspecies	Old Taxonomic Assignments	Current Taxonomic Reassignment	Coagulase
*S. aureus* subsp. *anaerobius* ^1^	*S. aureus*	*S. aureus* subsp. *anaerobius* later heterotrophic synonym of *S. aureus* subsp. *aureus.*	+
*S. carnosus* subsp. *utilis* ^2^	*S. carnosus*	*S. carnosus* subsp. *utilis* is later heterotypic synonym of *S. carnosus* subsp. *carnosus* ^2^	-
*S. cohnii* subsp. *urealyticus* ^3^	*S. cohnii*	*S. urealyticus* sp. nov.	-
*S. cohnii* subsp. *cohnii*	*S. cohnii*	*S. urealyticus* sp. nov.	-
*S. petrasii* subsp. *Jettensis*	*S. jettensis*	*S. petrasii* subsp. *jettensis* is a later heterotypic synonym of *S. petrasii* subsp. *petrasii.*	-
*S. fleurettii*	*S. sciuri*	*Mammaliicoccus fleurettii* ^4^	-
*S. lentus*	*S. sciuri*	*Mammaliicoccus lentus* ^4^	-
*S. stepanovicii*	*S. sciuri*	*Mammaliicoccus stepanovicii* ^4^	-
*S. sciuri*	*S. sciuri*	*Mammaliicoccus sciuri* ^4^	-
*S. vitulinus*	*S. sciuri*	*Mammaliicoccus vitulinus* ^4^	-
*S. schleiferi* subsp. *coagulans* ^5^	*S. schleiferi*	*S. coagulans* sp. nov.	-
*S. succinus* subsp. *casei* ^6^	*S. succinus*	*S. casei* sp. nov.	-
*S. petrasii* subsp. *pragensis* ^7^	*S. petrasii*	*S. pragensis* sp. nov.	-
*S. petrasii* subsp. *croceilyticus* ^8^	*S. petrasii*	*S. croceilyticus* sp. nov.	-

The recent reclassification of members of the family *Staphylococcaceae* by analyzing their core genome phylogeny complemented with genome-based indices and suggested the following proposals [2]: ^1^: The unification of *Staphylococcus aureus* subsp. *anaerobius* and *Staphylococcus aureus* subsp. *aureus* as *Staphylococcus aureus*. ^2^: The unification of *Staphylococcus carnosus* subsp. *utilis* and *Staphylococcus carnosus* subsp. *carnosus* as *Staphylococcus carnosus*. ^3^: *Staphylococcus cohnii* subsp. *urealyticus* as the novel species *Staphylococcus urealyticus*. ^4^: One clade, composed of *S. sciuri*, *S. fleurettii*, *S. lentus*, *S. stepanovicii*, *and S. vitulinus*, is deeply branching from the remaining *Staphylococcus* species and they are as equally distant from the other *Staphylococcus* species as *Macrococcus* is from *Staphylococcus*, it has been proposed that this clade be moved into the novel genus *Mammaliicoccus* with *Mammaliicoccus sciuri* as the type species. ^5^: *Staphylococcus schleiferi* subsp. *coagulans* as the novel species *Staphylococcus coagulans*. ^6^: *Staphylococcus succinis* subsp. *casei* as the novel species *Staphylococcus casei*. ^7^*: Staphylococcus petrasii* subsp. *pragensis* as the novel species *Staphylococcus pragensis*. ^8^: *S. petrasii* subsp. *croceilyticus* as the novel species *Staphylococcus croceilyticus*.

**Table 2:** Prevalence of methicillin-resistant *Staphylococcus aureus* (MRSA) in food-producing animals in different Arab countries.

Animals	Healthy/Sick	Samples	Prevalence of MRSA	Lineages of MRSA	Country	Reference
Cattle	Healthy	Nasal swabs	15.5%	N/D ^1^	Saudi Arabia	[52]
	Healthy	Nasal swabs	31%	N/D	Algeria	[53]
	Sick, mastitis	Milk samples	35.7%	N/D	Egypt	[54]
	Sick, mastitis	Milk samples	3.60%	ST4114-t10381, ST4120-t267	Tunisia	[55]
	Sick, mastitis	Milk samples	28.6%	CC1, CC5, CC45	Egypt	[56]
	Healthy	Milk samples	31.8%	N/D	Jordan	[57]
	Healthy	Nasal swabs	40%	N/D	Iraq	[58]
	Sick, mastitis	Milk samples	24.5%	CC5, CC22, CC88	Egypt	[59]
	Healthy	Nasal swabs, milk samples	3.7%	ST97-t267-agrI-SCC*mec*V	Tunisia	[60]
Sheep/goats	Healthy	Nasal swabs	9.3%	CC80-ST80	Algeria	[61]
	Healthy	Nasal swabs	3%	CC80-ST153-t044	Tunisia	[62]
	Healthy	Milk samples	29.8% sheep/11.5% goat	N/D	Jordan	[57]
	Healthy	Nasal swabs	28.9%	N/D	Saudi Arabia	[52]
	Healthy and Sick	Nasal swabs	2% (goats)	N/D	Saudi Arabia	[63]
	Healthy	Milk samples	17.14%	N/D	Saudi Arabia	[64]
Poultry	Healthy	Nasal swabs	30%	N/D	Algeria	[65]
	Healthy	swabs	27%	CC398; CC5	Egypt	[66]
	Healthy	Cloacal swab	27.3%	N/D	Iraq	[67]
	Healthy	Nasal swabs	Layers: 57%, broilers: 50%	N/D	Algeria	[53]
Camels	Healthy	Nasal swabs	4.4%	CC80	Algeria	[61]

^1^ N/D: Not Determined.

## Data Availability

Not applicable.
